# Autophagy-prominent cell clusters among human lens epithelial cells: integrated single-cell RNA-sequencing analysis

**DOI:** 10.1186/s12886-023-02910-8

**Published:** 2023-04-20

**Authors:** Jiasheng Liu, Mengchao Zhu, Yitong Xu, Mengdi Zhang, Haisen Sun, Yaqi Wang, Qingwen Yang, Jin Li

**Affiliations:** Department of Cataract, Eye Hospital of Wenzhou Medical University, 270# West Xueyuan Road, Wenzhou, 325000 Zhejiang China

**Keywords:** Human lens epithelial cells, Autophagy, Single-cell RNA-sequencing, Transmission electron microscopy

## Abstract

**Background:**

Autophagy is an important process that maintains the quality of intracellular proteins and organelles. There is extensive evidence that autophagy has an important role in the lens. Human lens epithelial cells (HLECs) play a key role in the internal homeostasis of the lens. HLEC subtypes have been identified, but autophagy-prominent cell clusters among HLECs have not been characterized.

**Purpose:**

To explore the existence of autophagy-prominent cell clusters in HLECs.

**Methods:**

Three donated lenses (HLECs from two whole lenses and HLECs from one lens without the anterior central 6-mm zone) were used for single-cell RNA sequencing (scRNA-seq). AUCell and AddModuleScore analysis were used to identify potential autophagy-prominent cell clusters. Transmission electron microscopy (TEM) was used to confirm the results.

**Results:**

High-quality transcripts from 18,120 cells were acquired by scRNA-seq of the two intact lenses. Unsupervised clustering classified the cells into four clusters. AUCell and AddModuleScore analysis revealed cluster 1 is autophagy-prominent. scRNA-seq analysis of HLECs from the lens capsule lacking the central zone confirmed the cluster 1 HLECs was located in the central capsule zone. The TEM result showed that greater autophagy activity was observed in the HLECs in central capsule zone, which further supported the above conclusions based on scRNA-seq analysis that autophagy was prominent in the central zone where the cluster 1 HLECs located.

**Conclusions:**

We identified an autophagy-prominent cell cluster among HLECs and revealed that it was localized in the central zone of the lens capsule. Our findings will aid investigations of autophagy in HLECs and provide insights to guide related research.

## Introduction

Autophagy is an evolutionarily conservative and lysosomal-dependent catabolic process that degrades damaged organelles, protein aggregates, and lipid droplets; it subsequently recycles the degraded components [1]. Three types of autophagy have been identified: macroautophagy, chaperone-mediated autophagy, and microautophagy [2]. Among these three types, macroautophagy (hereafter, “autophagy”) is the most widely studied and most easily recognized process [3].

Autophagy in the lens has been extensively studied [4–6]. Zhang et al. reported that the upregulation of autophagy-related gene 4 A inhibited apoptosis in human lens epithelial cells (HLECs)[7]. Additionally, there is evidence that the maintenance of autophagy activity is important to prevent aging in HLECs [8]. Oxidative stress triggers persistent activation of autophagy during aging in HLECs, leading to excessive degradation of p62 protein and the onset of premature aging [9]. However, studies of lens autophagy have typically used HLEC lines or HLECs isolated from human lenses, which were studied without distinction among cell subtypes. Recently, distinct subtypes were identified in HLECs [10]. Thus, there is a need to investigate the potential presence of autophagy-prominent cell clusters among HLECs.

In this study, we analyzed three donated human lenses to identify potential autophagy-prominent cell clusters among HLECs. scRNA-seq analysis of HLECs from the lens capsule lacking the central zone confirmed the cluster 1 HLECs was located in the central capsule zone. The TEM result showed that greater autophagy activity was observed in the HLECs in central capsule zone, which further supported the above conclusions based on scRNA-seq analysis that autophagy was prominent in the central zone where the cluster 1 HLECs located.

## Materials and methods

### Clinical samples

Human lenses were provided by the Eye Bank of Wenzhou Medical University. In total, three donated lenses were used for scRNA-seq (Table 1). Two lenses (5Y and 79Y) were subjected to scRNA-seq analysis of HLECs from the whole lens capsules; another lens (55Y) was subjected to scRNA-seq analysis after the central 6 mm anterior capsule torned. Seven donated lenses (16 M, 8Y, 17Y, 41Y, 46Y, 51Y and 67Y) were used for TEM sample preparation and observation. This study protocol was approved by the Human Ethics Committee of the Laboratory of Wenzhou Medical University.

**Table 1:** Each sample’s sequencing quality and detailed information

Sample	Age	Sex	Cell number	Mean reads per cell	Media genes per cell
1	5Y	M	10,811	76,978	3355
2	79Y	M	7309	127,828	3196
3	55Y	M	4651	143,528	1134

### scRNA-seq

#### Single-cell suspension preparation and sequencing

Separated the capsule from the lens,then lens capsules were digested in 600 µL of digestion solution (400 µL pancreatin, 100 µL collagenase A, and 100 µL dispase II) for 8 min at 37 °C; gentle pipetting was performed 20 times to assist cell dissociation. Subsequently, 600 µL of Dulbecco’s modified Eagle’s medium (containing 10% fetal bovine serum) were added to neutralize the digestion reaction. After neutralization, the capsule was removed; the remaining liquid was passed through a 40-µm filter. The resulting single-cell suspension was centrifuged at 1200 rpm for 5 min, the supernatant was discarded, and the cell pellet was resuspended in 1150 µL of phosphate-buffered saline solution. The single-cell suspension was immediately sent to 10x Genomics (CapitalBio Technology, China) for scRNA-seq analysis.

#### Exploration of scRNA-seq data

Reads were aligned and unique molecular identifier counts were obtained using the CellRanger pipeline (10x Genomics). Subsequent processing, integration, and downstream analysis were performed using R software (version 4.1.1) and Seurat software (version 4.1.0) [11]. Cells were filtered according to the numbers of genes and reads; genes with differential expression between samples were identified using the FindMarkers function. The autophagy score was calculated using the Seurat function AddModuleScore. Two autophagy databases, Human Autophagy Database (http://www.autophagy.lu/index.html) and HAMdb (http://hamdb.scbdd.com/)[12], were used for AddModuleScore analysis. For comparison of autophagy pathway activity among individual cells, autophagy pathway data were retrieved from the Kyoto Encyclopedia of Genes and Genomes (http://www.genome.jp/kegg/) [13], then subjected to assessments using the R package AUCell [14].

#### TEM

The zone from the anterior pole of the lens to half the distance from the equator was divided into a central zone and a non-central zone; the non-central zone included a peripheral zone and an equatorial zone. Lens sections (approximately 200 μm thick) were cut from the fresh lenses using a thin oscillating knife, then divided into a central zone and a non-central zone. The sections were fixed for 12 h in 0.1 M phosphate buffer (pH 7.4) containing 2% paraformaldehyde and 2% glutaraldehyde. After removal of the fixative, lens samples were washed three times with phosphate-buffered saline for 15 min each, treated with 1% osmic acid for 60 min, washed three times with deionized distilled water for 15 min each, dehydrated in an acetone series (30%, 50%, 70%, 80%, and 90%) for 15 min per concentration, and finally incubated for 15 min in 100% acetone. Lens tissues were embedded in Eponate 12 resin, then used to prepare semi-thin and ultra-thin sections. For ultra-thin sections, 70–90-nm sections were collected on 200-mesh grids, stained with 5% uranyl acetate and 0.3% lead citrate, and visualized using an H-7500 transmission electron microscope operating at 60 kV.

### Statistical analysis

#### scRNA-seq

Statistical analysis was performed by two-sided Wilcoxon rank-sum tests, followed by Benjamini–Hochberg correction.

#### TEM

Statistical analysis was carried out using SPSS software (version 26.0, IBM Corp.). Each data set was subjected to normality assessment using the Kolmogorov–Smirnov normality test. All data exhibited a normal distribution; thus, they were expressed as means ± standard deviations. Comparisons between two groups were performed using unpaired Student’s t-tests.

## Results

### Single-cell transcriptome profiles revealed different subtypes of HLECs

**Fig. 1:**
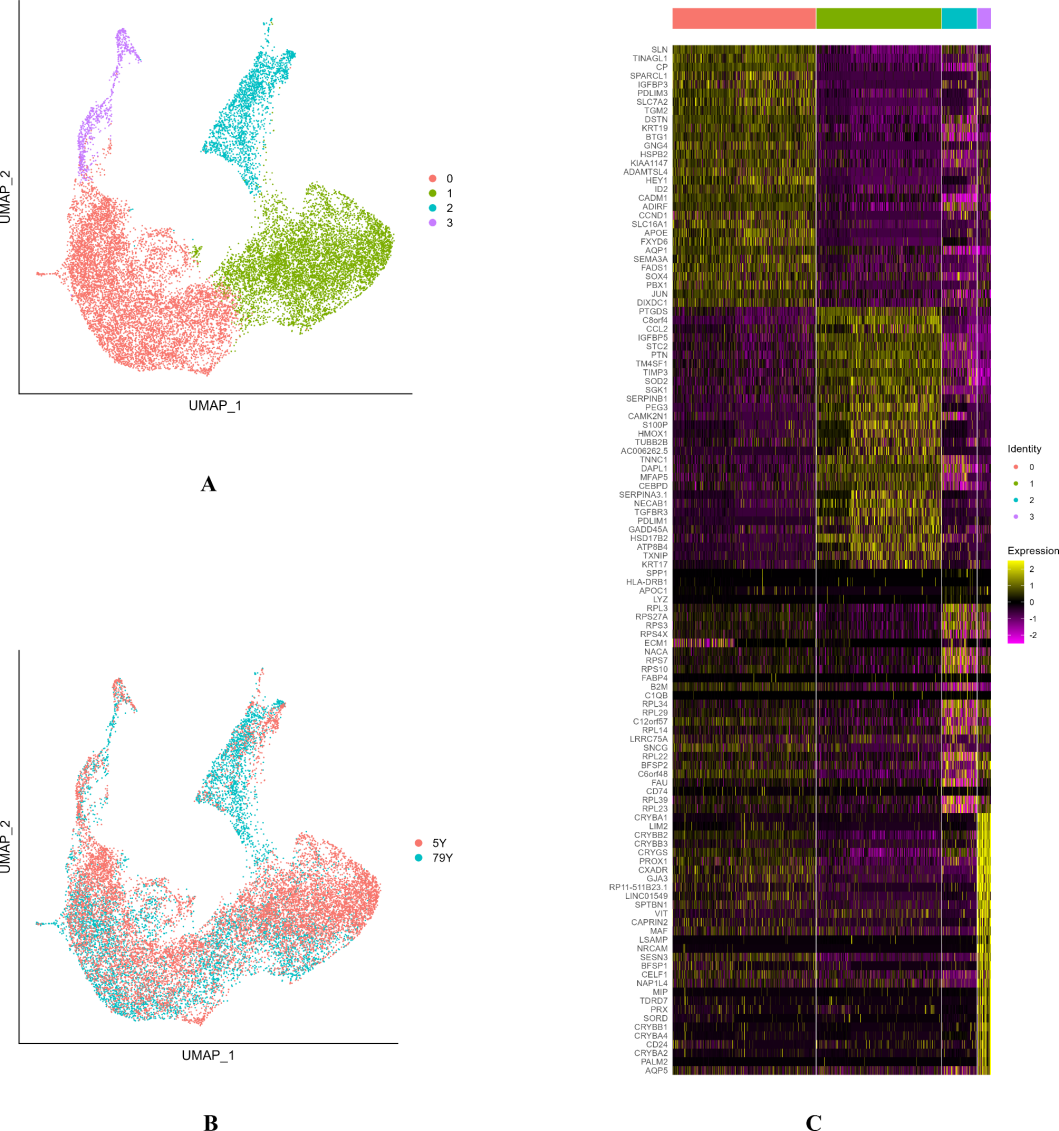
**A**: UMAP of HLECs from two donor tissues generated single-cell transcriptomic profiles of total 18,120 cells with 4 differential cell clusters .**B**: The distribution of two samples in the cluster. **C**: correspond to the top 30 differential genes in the respective HLEC clusters 0 to 3

High-quality transcripts from 18,120 cells were acquired by scRNA-seq of the two intact lenses using the 10x Genomics platform. Unsupervised clustering analysis in Seurat revealed four cell clusters, which were visualized by uniform manifold approximation and projection embedding (Fig. 1A、B). Distinct transcriptome profiles were identified in each of the four cell populations, as shown in the representative diagrams of the top 30 differentially expressed genes from each cluster (Fig. 1C).

### Autophagy score of each cluster

**Fig. 2:**
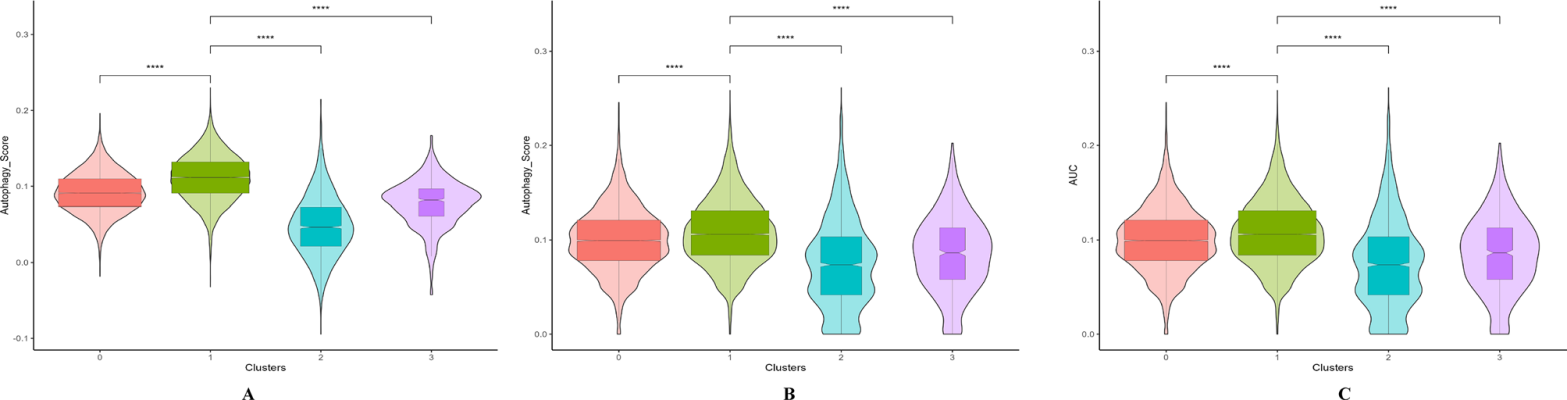
Autophagy scores. **A**: Autophagy score using Human Autophagy Database. **B**: Autophagy score using HAMdb. **C**: AUCell scores of autophagy signaling pathways in each of the four cell clusters. ****adj. p-value < 0.0001

To identify a potential autophagy-prominent cell cluster, we compared autophagy scores among clusters. AddModuleScore analysis using two autophagy databases revealed that the autophagy score of cluster 1 was significantly higher than the autophagy scores of the other three clusters (Fig. 2A, B). AUCell analysis also showed that the autophagy signaling pathway in KEGG had the highest score in HLEC cluster 1 (Fig. 2C).

### Location of autophagy-prominent cell cluster

**Fig. 3:**
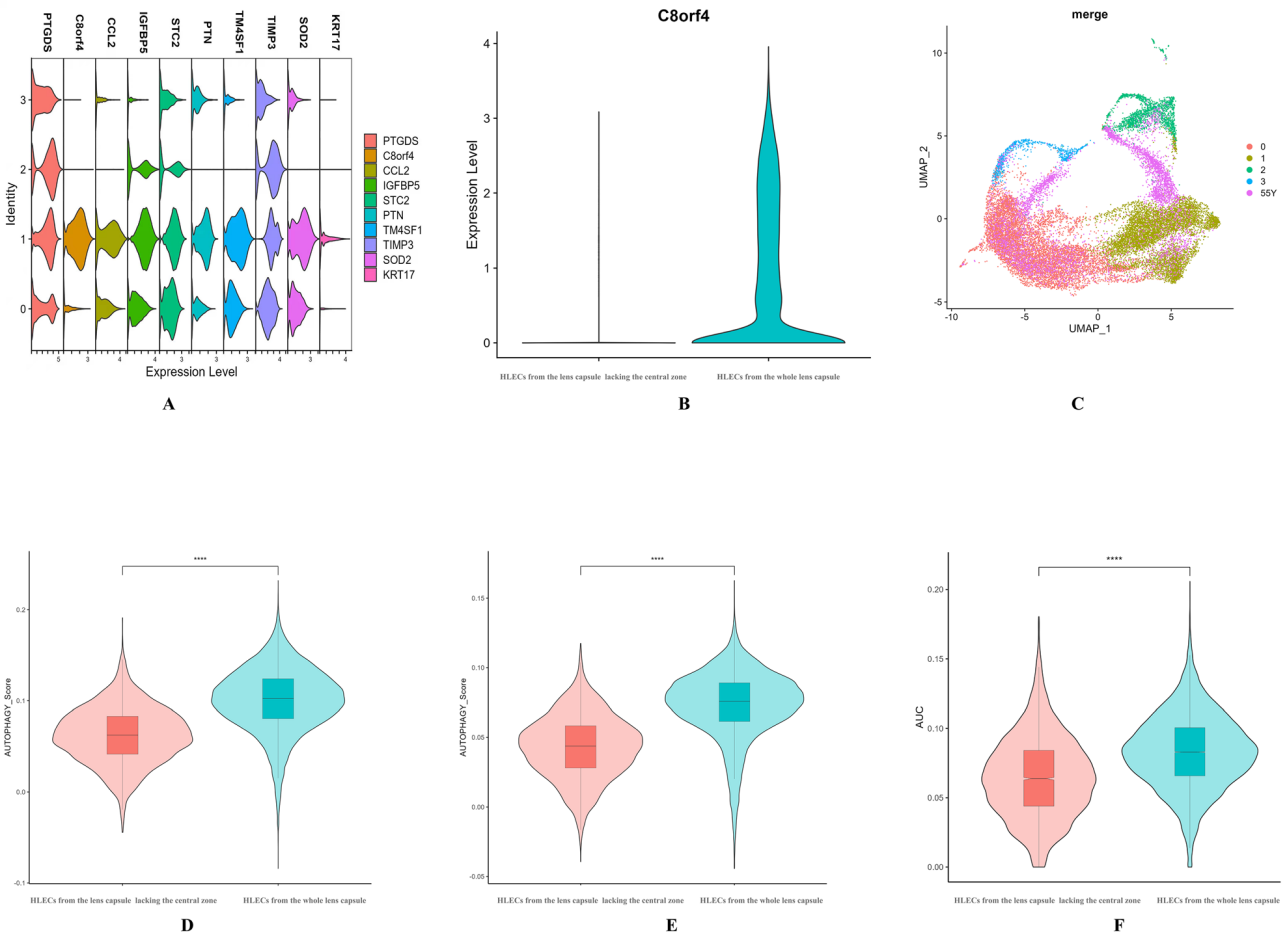
**A**: Violin plot of the top 10 differential genes in cluster 1. **B**: Expression of C8orf4 in HLECs from the lens capsule lacking the central zone and HLECs from the whole lens capsule. **C**: Map the dataset of HLECs from the lens capsule lacking the central zone to the dataset of HLECs from the whole lens capsule. **D**: Autophagy score using Human Autophagy Database. **E**: Autophagy score using HAMdb. **F**: AUCell scores of autophagy signaling pathways. ****adj. p-value < 0.0001

The TOP10 specific expression gene map of cluster 1 (Fig. 3A) revealed that the marker C8orf4 consistently distinguished cluster 1 from the other clusters. C8orf4, also known as thyroid cancer-1 (TC-1), was originally identified as a highly expressed gene in papillary thyroid carcinoma and surrounding normal thyroid tissues [15]. C8orf4 is ubiquitous in vertebrates and encodes a 106-amino acid protein without a clear functional domain [15–17]. Compared with HLECs from the whole lens capsule, HLECs from the lens capsule lacking the central zone almost did not express C8orf4 (Fig. 3B). The mostly missing of cell cluster 1 was found after mapping the dataset HLECs from the lens capsule lacking the central zone to the dataset of HLECs from the whole lens capsule(Fig. 3C).AddModuleScore analysis using two different autophagy databases revealed that, compared with HLECs from the lens capsule lacking the central zone, HLECs from the whole lens capsule had a higher autophagy score (Fig. 3D, E). AUCell scored autophagy through the autophagy pathway of KEGG, and the results were also consistent with the above (Fig. 3F).

### Comparison of autophagy between central and non-central zones of HLECs via TEM

**Fig. 4:**
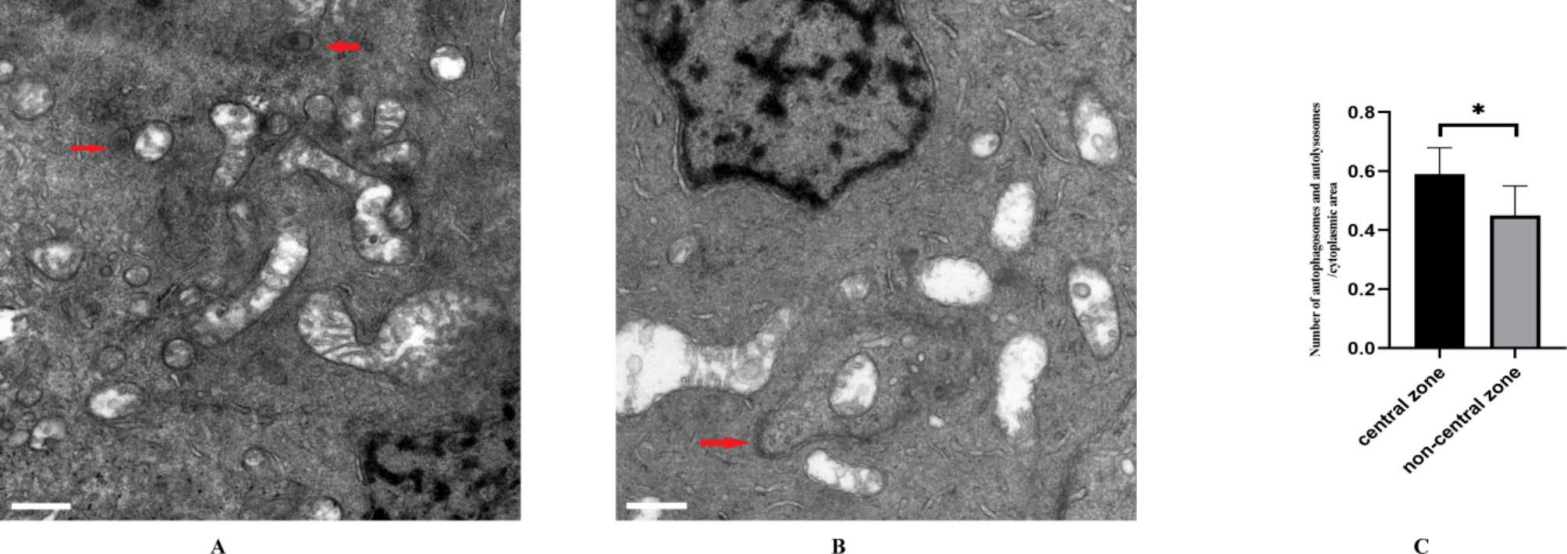
Autophagic vacuoles in central and non-central zones of HLECs (n ≥ 70 cells from seven lenses). **A**: 67Y, central zone. **B**: 67Y, non-central zone. Red arrows: autophagic vacuoles. Scale bars in A and B = 0.5 μm. Data in the graph are shown as means ± standard deviations; p-values < 0.05 were considered statistically significant

To determine whether HLECs in the central zone exhibited greater autophagy activity, we used TEM to compare autophagic vacuoles between central and non-central HLECs. Under different random fields of view and different degrees of magnification, greater autophagy activity was observed among HLECs in the central zone. The numbers of autophagic vacuoles per 10-µm^2^ cytoplasmic zone were 0.59 ± 0.09 (central zone) and 0.49 ± 0.1 (non-central zone) (p < 0.05) (Fig. 4A–C). Thus, TEM findings supported the conclusions based on scRNA-seq analysis.

## Discussion

In this study, we used scRNA-seq analysis of HLECs to identify a potential autophagy-prominent cell cluster and reveal its location in the central zone of the lens. TEM-based comparison of the numbers of central and non-central autophagic vacuoles supported the conclusions based on scRNA-seq analysis. The presence of autophagy-prominent cell clusters is consistent with the notion that autophagy in HLECs cannot be regarded as a single process. These findings indicate that a relationship is present between autophagy and HLECs in different locations; they provide new insights for analyses of autophagy in HLECs.These findings indicate that a relationship is present between autophagy and HLECs in different locations; they provide new insights for analyses of autophagy in HLECs.

Autophagy can control intracellular quality by removing damaged organelles and protein aggregates [1, 18]. There is evidence that autophagy is necessary for lens function; autophagy dysfunction may lead to the loss of stress resistance within the lens [19]. HLECs proliferate into fiber cells, which comprise a large portion of the lens throughout life [6, 20, 21]. HLECs play a key role in maintaining internal homeostasis within the lens [22]. The maintenance of a dynamic balance between “intracellular waste production” and autophagy is important for HLEC homeostasis and lens physiology.

Here, we found that autophagy was prominent in cluster 1.we maped the HLECs from the lens capsule lacking the central zone to the HLECs from the whole lens capsule, and really found that the cluster 1 is mostly missing in the former. The single-cell data showed that compared with HLECs from the whole lens capsule, HLECs from the lens capsule lacking the central zone rarely expressed C8orf4 which is the special marker of cluster1. The TEM result showed that greater autophagy activity was observed in the HLECs in central capsule zone, which further supported the above conclusions based on scRNA-seq analysis that autophagy was prominent in the central zone where the cluster 1 HLECs located.

We speculate that an autophagy-prominent cell cluster exists in the central zone because, compared with the non-central zone, the central zone of the lens is more easily exposed to ultraviolet radiation. Greater exposure to ultraviolet radiation leads to enhanced production of reactive oxygen species. Autophagy can delay cell death by clearing damaged mitochondria, endoplasmic reticulum components, peroxisomes, and proteins damaged by oxidative stress; this clearance can effectively eliminate oxidative stress-related damage, thereby maintaining cellular homeostasis [23]. In the non-central zone, sufficient autophagy activity can maintain cell homeostasis and viability. Conversely, in the central zone, autophagic flux and waste production may not achieve an efficient dynamic balance; because of the greater damage exposure, this zone may contain a cluster of autophagy-prominent cells.

A limitation of this study is that our samples come from donated lenses. Due to the difficulty of lens collection, the donated lens used in this study did not choose the same age lens, and the age factor may affect the localization of autophagy clusters.

## Conclusion

In conclusion, we determined the presence and location of an autophagy-prominent cell cluster among HLECs, which may provide new insights into autophagy within the lens.

## Data Availability

The datasets generated and/or analysed during the current study are available in the GitHub repository, https://github.com/tiger1916/lens-scRNA.
